# Measurement of Tissue Oximetry in Standing Unsedated and Sedated Horses

**DOI:** 10.3390/vetsci8100202

**Published:** 2021-09-22

**Authors:** Nicholas Cowling, Solomon Woldeyohannes, Albert Sole Guitart, Wendy Goodwin

**Affiliations:** Gatton Campus, School of Veterinary Science, The University of Queensland, Gatton 4343, Australia; nicholas.cowling@uqconnect.edu.au (N.C.); s.woldeyohannes@uq.edu.au (S.W.); a.guitart@uq.edu.au (A.S.G.)

**Keywords:** horse, tissue oximetry, near infrared spectroscopy, sedation

## Abstract

Near infrared spectroscopy (NIRS) noninvasively measures peripheral tissue oxygen saturation (StO_2_) and may be useful to detect early changes in StO_2_ in anaesthetized and critically ill horses. This study aimed to identify the muscle belly that provided the highest percentage of successful StO_2_ readings and the highest mean StO_2_ value. Fifty adult horses were enrolled in a prospective controlled study. StO_2_ was measured at six different muscles in each horse, for each intervention: hair overlying the muscle was clipped (post clipping: PC), clipped skin was cleaned with chlorhexidine (post-surgical prepping: PP) and medetomidine was administered intravenously (post medetomidine: PM). Mean StO_2_ values were calculated for each muscle, and a linear effects model was used to assess the effect of muscle group and intervention on StO_2_. The sartorius muscle gave the highest percentage of successful StO_2_ values (*p* < 0.001) and the highest mean (90% CI) StO_2_ values for the PC, PP and PM interventions. Surgical prepping of the skin increased the success for measurement of StO_2_ values. For all muscles, administration of medetomidine was associated with lower StO_2_ values (*p* < 0.001). In conclusion, of the muscles examined, the sartorius muscle may be the preferred muscle to measure StO_2_ in horses, and clipping and cleaning of the probe placement site is recommended.

## 1. Introduction

Tissue oxygen saturation (StO_2_) as measured by Near Infrared Spectroscopy (NIRS) is a novel, non-invasive, monitoring modality that measures the oxygen saturation of hemoglobin in the tissues. Tissue oxygen saturation assessment utilizes the increased permeability of skin and tissue to near infrared (NIR) light and allows for the determination of oxygen saturation at greater depths than pulse oximetry [1,2]. Similar to pulse oximetry, NIRS relies on the differential absorption of specific wavelengths of light by oxyhemoglobin and hemoglobin [3,4]. The Modified Beer Lambert Law is then used to quantify the oxygen saturation of red blood cells in the tissue, with the monitor analyzing both the arterioles and venules with a weighting of 30 and 70%, respectively [5].

Experimental studies in dogs and pigs have found a significant correlation between StO_2_ and changes in global oxygen delivery (ḊO_2_) [6,7,8]. Clinically, the technology is currently used in human healthcare to monitor StO_2_ in patients suffering from sepsis, patients undergoing cardiopulmonary bypass, and in pediatric patients to assess hypoxic ischaemia in the peri parturient period [9,10,11,12,13,14]. Theoretically, early detection of changes in StO_2_ as an indirect measurement of ḊO_2_ may allow for early, goal-directed interventions to improve perfusion and oxygen delivery to the tissues.

In horses, general anaesthesia is often associated with impaired pulmonary function and resultant hypoxemia. The use of a non-invasive monitor, such as NIRS, that provides continuous, reliable assessment of ḊO_2_ and responds rapidly to changes in perfusion could offer a significant improvement in perioperative monitoring of anaesthetized horses. Currently, there is limited information regarding the use of NIRS to measure StO_2_ in horses. A study investigating a no-longer commercially available NIRS monitor in standing, un-sedated horses reported that the vastus lateralis muscle and extensor carpi ulnaris muscle gave the most reliable and repeatable StO_2_ readings [15]. Of note, reference intervals for StO_2_ are not interchangeable between monitors, with human studies reporting differences between the normal reference intervals of different monitors as high as 13% [16,17].

The INVOS 5100c tissue oximeter (Medtronic, Minneapolis, MN, USA) is a two-wavelength, dual-receiving optode oximeter that can simultaneously monitor StO_2_ or cerebral oximetry (rSO_2_) at up to four different locations on the body [18,19,20]. The monitor is commercially available and has previously been used by McConnell et al. [21] to monitor rSO_2_ in horses.

The primary aim of this study was to identify the muscle that gave the highest percentage of successful StO_2_ readings and identify the muscle that gave the highest mean StO_2_ values, in healthy, standing, un-sedated horses using the INVOS 5100c monitor. A secondary aim was to identify any changes in StO_2_ following two interventions (cleaning of the skin and administration of medetomidine).

## 2. Materials and Methods

### 2.1. Animals

Fifty (50) horses owned by the University of Queensland were enrolled in the study. All horses were deemed to be healthy on the day of the study following examination by a veterinarian. Animals with superficial wounds or infections over the proposed StO_2_ probe application sites were excluded from the study. A variety of horse breeds were included in the study: Standardbreds (31/50, 62%), Thoroughbreds (8/50, 16%), Australian stock horses (7/50, 14%), Warm Blood crosses (3/50, 6%) and an Andalusian (1/50, 2%). Horse coat colors included: bay (22/50, 44%), brown (14/50, 28%), dark brown (2/50, 4%), chestnut (3/50, 6%), grey (5/50, 10%), dunn (1/50, 2%) and black (3/50, 6%). Eighty-two percent of these were classified as heavily pigmented. The study was reviewed and approved by the University of Queensland Animal Ethics Committee (AEC # AE36185).

### 2.2. Experimental Protocol

Horses were held in yards located near the stocks prior to initial handling.

Prior to treatment, horses were weighed and moved into individual stocks and allowed a 5-min acclimatization period. A physical examination was then performed and included temperature, heart rate (HR), cardiac auscultation, respiratory rate (f_R_), capillary refill time (CRT) and peripheral examination of the muscular skeletal system.

Six muscle bellies were selected for application of the probe of the INVOS 5100c StO_2_ monitor and included sartorius, biceps brachii, semimembranosus, extensor digitorum longus (EDL), brachiocephalicus (BRACH) and extensor carpi radialis (ECR). The muscle bellies were selected for the following reasons:

(i) Success in other species such as the sartorius muscle in dogs [22];
(ii) Potential ease of access and probe maintenance during recumbency during general anaesthesia. For example, the ECR is easily accessible in animals in dorsal or lateral recumbency;
(iii) Presumed thin skin and minimal hair covering that may allow for more successful readings.

The left side of the horse was used for the assessment of each of these muscle bellies. The hair over the selected muscle bellies was clipped with a size 40 surgical blade.

The StO_2_ probe was held over the clipped area for 1 min, with a flush application of the probe to the skin to exclude external light sources. StO_2_ and the signal-strength index (SSI) values were recorded at 20 s intervals for 1 min, with a total of three values recorded. If no reading was attained at any 20 s interval, it was recorded as no signal for both StO_2_ and SSI. The probe was moved to the area over the next muscle belly and the process repeated. The order for probe placement was: sartorius, biceps brachii, semimembranosus, EDL, ECR and BRACH muscle.

After initial readings, the clipped skin over the muscles was cleaned using a soapy chlorhexidine solution (Chlorhex-S, Jurox PTY LTD, Rutherford, Australia) until the swabs showed no visible dirt. The skin was then wiped with a water-soaked swab to remove residual chlorhexidine. The probe was placed as previously described and values were recorded.

Blood was collected for evaluation of packed cell volume (PCV) and total protein (TP). Medetomidine (Medetomidine, Troy Laboratories Australia Pty Ltd., Glendenning, Australia) was then administered to the horses at 7 μg kg^−1^ IV. Five minutes later, HR and f_R_ were recorded and StO_2_ readings commenced as previously described.

This study resulted in three intervention groups for StO_2_ measurement: post clipping (PC), post-surgical prepping (PP) and post medetomidine (PM). Following completion of data collection, horses were held in the stocks for approximately 30 min to allow for the sedative effects to wane. Horses were then moved back to the holding yards for at least another 2 h, where they were monitored for signs of residual sedation before being returned to their paddocks.

### 2.3. Statistical Analysis

A power analysis was performed and determined that a sample size of 48–54 horses was required to provide 80–90% power [15,23]. Descriptive summary statistics were used to summarize horse characteristics, and normal distribution of StO_2_ values was checked using the Shapiro–Wilk test. Zero values were excluded from the statistical analysis as they represent a failure of the machine to receive enough NIR light back from the tissues and do not represent a low StO_2_ value. For normally distributed StO_2_ values, parametric reference intervals were computed, and for those non-normally distributed StO_2_ values, non-parametric reference intervals were calculated.

Tests of homogeneity of successful readings between muscle groups and pair-wise comparisons were performed. To compare the proportions of successful readings between muscle groups, a test of homogeneity of proportions between groups was conducted. Similarly, homogeneity and pair-wise comparisons (pre-clipping (PC) is considered as a reference group in the comparison) were performed for comparing the proportion of successful readings among the intervention groups (pre-clipping (PC), post prepping (PP) and the post medetomidine (PM)). In addition, a linear mixed-effects model (LMM) was fitted to assess the effect of the treatment group (PC, PP and PM) on StO_2_, utilizing each horse as the experimental unit. All analyses were conducted in R, Package Version 1.2.0 (The R Foundation, Vienna, Austria, http://www.R-project.org, accessed on 20 April 2020). The Reference Intervals R package was utilized to find reference intervals for StO_2_ in standing horses (Reference Intervals R, R Foundation for Statistical Computing, Vienna, Austria, http://www.R-project.org, accessed on 20 April 2020).

## 3. Results

### Horse Characteristics

Fifty horses were included in the analysis with descriptive statistics for weight, sex, PCV and TP shown in Table 1.

Summary statistics for total number of successful readings and percentage number of successful readings for intervention and muscle groups are shown in Table 2. The monitor failed to display any values, for any muscle, in all three treatment groups for two brown horses (2/14). One horse strongly resented the placement of the StO_2_ probe for 5 out of 6 muscles when attempting to attain readings for the PP group. The skin over the sartorius was not able to be clipped due to its location in the inguinal region of the horse. Clipping of the sartorius was attempted on the first three horses; after strong and persistent resentment, this was abandoned. There was a significant difference in the number of successful readings between the PC and PP groups in all muscles except for the digitorum longus (*p* < 0.05).

Summary statistics for measured StO_2_ values are shown in Table 3. StO_2_ values were normally distributed for each muscle and treatment except for the PM biceps brachii and the EDL, which did not have enough values to assess normalcy. No significant difference in StO_2_ values was found between the PC and PP interventions; however, as noted in Table 2, there was an increase in the number of successful StO_2_ readings following PP. There was a significant (*p* < 0.05) decrease in StO_2_ between the PP and PM interventions for the following muscles groups: Biceps Brachii (*p* = 0.001), BRACH (*p* = 0.0010), ECR (*p* < 0.0001), Semimembranosus (*p* = 0.0258) and Sartorius (*p* < 0.0001).

Results of the linear mixed effects model (LMM) are shown in Table 4. For modelling, the PC intervention, and the biceps brachii muscle were used as the reference intervention group and muscle, respectively. Overall, administration of medetomidine (PM) had a negative effect on StO_2_ with values 9.2% less than the reference group. With regards to the muscle group, the sartorius muscle was positively associated with StO_2_, with values for this muscle 5% higher than the refence muscle, independent of intervention. All other muscles had a negative association with StO_2_ when compared to the reference muscle, independent of treatment. The interhorse variability of StO_2_ was 38.25%, and the intraclass correlation was found to be 0.43.

## 4. Discussion

This study demonstrated that the INVOS 5100c tissue oximeter was capable of measuring StO_2_ in standing horses, with varying success in the muscles investigated. The sartorius, ECR and BRACH muscles had the highest, most consistent StO_2_ readings in the standing horses. Analysis revealed a wide reference interval for each muscle, reinforcing previous research that suggests that StO_2_ should be used as a trend monitor rather than assessing absolute values. Finally, it demonstrated that the administration of a clinically appropriate dose of medetomidine significantly reduced the StO_2_ in all muscles examined except for the EDL.

Critically ill, anaesthetized horses are usually positioned in dorsal recumbency. Consequently, when deciding on muscles for probe location, a major consideration was selecting a region that could be easily accessed while horses were in dorsal recumbency. As a result, muscles selected included the semimembranosus, EDL, biceps brachii, ECR and BRACH muscles. This study identified that surgical preparation of skin clipped of hair improved the ability of the machine to attain StO_2_ readings, as demonstrated by the PP intervention having a higher number of successful readings when compared to readings taken after clipping only (PC). Additionally, readings taken during the PM intervention, despite the significant decrease in StO_2_ values, had the second-highest number of successful readings. Surgical preparation of the skin was performed, as it was hypothesized that hair and dirt would interfere with the passage of NIR light. Previous investigations of NIRS technology in the horse have accounted for the potential interference of hair by clipping the regions for probe placement; however, this is the first study to undertake skin preparation prior to probe placement [15,21].

When muscle bellies were prepared in this way, the sartorius resulted in the greatest number of successful readings, with the ECR and BRACH muscles being the next most successful. An overall high variability in percentage of successful readings was noted, which is different from the findings of Gingold et al. [15]. The study reported a range of successful readings between 70–86% for the five muscles examined, with those muscles having a similar preparation to the PC group in the current study. Likely contributing to the difference in success rate between the present study and the Gingold et al. research is the employment of a different NIRS StO_2_ monitor, as well as investigation of a number of different muscle bellies. In several previous publications, it has been shown that different monitors gave different StO_2_ values, even when applied to the same patient and the same muscles. For example, Engbers et al. (2014) found a mean StO_2_ difference in the dog of 23% when comparing the INVOS 5100c to the InSpectra. This variation between monitors is thought to be related to both the number of NIR wavelengths utilized and the internal algorithms used to calculate the values.

The effect of coat color on success rate of StO_2_ values was identified by Gingold et al. [15], where it was noted that light colored horses had a higher percentage of successful StO_2_ readings compared with dark and medium-colored ones. This may be explained by correlating dark coat colour with increased skin pigmentation. Melanin is a chromophore, like hemoglobin, that can absorb NIR light and thus increasing levels have been noted to affect the accuracy and ability for StO_2_ monitors to obtain readings [24]. Given that 81% (41/50) of the horses in the present study were considered to have increased pigmentation (brown, bay and black coat colour), this could also account for the significant difference in success rate when compared with the Gingold et al. [15] findings, where there was a higher proportion of lighter-colored horses (7/30 light-colored; 21/30 medium-colored; 2/30 dark-colored).

The six muscle bellies examined had mean StO_2_ values calculated across each of the three intervention periods. The sartorius achieved the highest value during the PP treatment period. During this treatment, the next-highest mean recorded was in the biceps brachii and BRACH. However, all three muscles had mean values considerably lower than those reported by Gingold et al. [15], where the mean StO_2_ (CI 90%) of the vastus lateralis and the extensor carpi ulnaris in horses was 95% (93.8–96.5) and 93% (91.6–93.9), respectively. These values were up to 50% higher than found in the current study. Again, it is hypothesized that differences in monitor technology, as discussed previously, as well as muscle utilization are key factors in explaining the discrepancy in mean StO_2_ values.

Another factor known to affect StO_2_ readings is body condition score (BCS) and adipose tissue, as documented in both dogs and humans [22,25,26]. Increasing tissue thickness is an issue in reflectance mode NIRS, as the light can only penetrate the same distance that the optodes are apart [18]. Unfortunately, the BCS of the horses examined in this study was not recorded; therefore, it is not possible to comment on whether adipose tissue may have influenced the results.

Wide confidence intervals for the mean StO_2_ were calculated, and further analysis found a high degree of interhorse variability. Multiple reports in the human field have also noted interpatient variability in both healthy volunteers and in patients with sepsis, with reports indicating up to 10% variation [19,27,28]. One explanation for the high interhorse variation is age. Lian et al. [29] noted that elderly patients were more likely to produce lower StO_2_ values when compared with other age groups. Although this association was not assessed in the present study, it is plausible that age may also have factored into the interhorse variability. Ultimately, the high interhorse variability noted in the current study supports previous research that the StO_2_ monitor should be utilized for interpreting trends rather than absolute sole values [30,31]. This suggests that rather than relying on reference ranges to help interpret StO_2_ values, a more sensical system would be to treat StO_2_ values as unique to individuals. Such a system would factor in relative decreases in StO_2_ values from the individual’s baseline in order to initiate interventions.

The alpha 2 (α2) adrenergic agonist drugs are among the most common sedatives used by veterinarians to enable the handling and treatment of horses. This class of drug results in a reliably sedated patient but can cause significant cardiovascular abnormalities. The use of medetomidine in the equine veterinary field is increasing as a greater number of veterinarians realize its benefits over xylazine [32]. Despite the greater specificity for the α2 receptor, negative effects are still seen, such as decreases in CO and ḊO_2_ [33,34,35,36]. It was reasoned that the alteration in ḊO_2_ caused by medetomidine would be useful in the current study to assess if NIRS technology could detect potential decreases in ḊO_2_, as evidenced by lower StO_2_ values. The dose that was selected was a clinically appropriate dose and thus is relevant to clinical considerations [37]. It was found that medetomidine (PM group) had significant effect on mean StO_2_ values, causing a significant decrease (ranging from 8–12% reduction) when compared to the PP values. This excluded the EDL, where there were insufficient values to access for normalcy.

It is postulated that this decrease in StO_2_ post medetomidine administration was due to a decrease in both CO and ḊO_2_. Pavlisko et al. [6,7] found similar results when they experimentally created conditions of low CO and low ḊO_2_ in anaesthetized dogs, reporting a strong correlation with decreases in StO_2_. We cannot definitively conclude that CO and ḊO_2_ decreased in response to medetomidine and thus decreased StO_2_, however it seems a reasonable assumption. Consequently, the monitor may have the potential to detect clinically relevant alterations in StO_2_ and allow for early intervention. This would be particularly useful during anaesthesia where general anaesthesia is often associated with impaired pulmonary function, resultant hypoxemia and presumably a reduction in ḊO_2_ [38,39].

This study had several limitations. Firstly, the authors failed to record either BCS or the age of the horse subjects, two parameters that previous studies have identified may affect StO_2_ values. Secondly, given the affect coat color had on StO_2_ values, it would be beneficial to have a more normally distributed number of animals across the three coat categories in order to better account for any affect melanin might have on values. Additionally, by not measuring ḊO_2_ or PaO_2_, it is difficult to definitively link StO_2_ and global oxygen delivery in horses, which would be of great benefit, given that similar links have been established in both the human and canine model. Finally, the muscle for the StO_2_ probe location was chosen with consideration for ease of access when horses were anaesthetized and positioned in dorsal recumbency. It was reasoned that this was the position that most critically ill animals would be positioned in for emergency surgery, and thus the muscles selected included the semimembranosus, EDL, biceps brachii, ECR and BRACH muscles. In retrospect, it may have also been useful to examine muscles that were likely to undergo excessive compression during anaesthesia, to potentially monitor local StO_2_.

Future studies investigating StO_2_ in horses should consider the utilization of a five-wavelength monitor to counteract pigmentation confounding StO_2_ values or aim to have a large sample size for each coat category. Furthermore, studies should consider the potential for NIRS application for anaesthetized patients and critical care patients such as acute abdomen equine colic cases and other equine anaesthesia. A key requirement for this would be foundational research that aimed to determine whether there was a correlation between decreases in ḊO_2_ and StO_2_. The establishment of an intervention point for StO_2_ would also be valuable; for example, an StO_2_ below 35% would represent a significant decrease and would necessitate the application of a treatment. This would allow for the development of treatment algorithms for managing decreased StO_2_.

## 5. Conclusions

This research was the first to identify the muscle locations that gave the most consistent and highest StO_2_ reference intervals using the INVOS 5100c for StO_2_ in standing horses. The monitor was able to attain StO_2_ values from horses of a variety of colours and sexes, though it was postulated that pigmentation likely reduced the ability of this two-wavelength monitor to attain StO_2_ values. Additionally, surgical preparation of the skin will likely improve the oximeters’ ability to attain readings. The sartorius muscle was found to provide the most consistent and reliable readings. However, as accessing this muscle in the standing horse was not practical, the authors recommend the use of the ECR or BRACH muscle.

## Figures and Tables

**Table 1:** Summary statistics of the age, weight, packed cell volume (PCV) and total protein (TP) for 50 horses.

Summary Stats					
	Median	IQR	Min	Max	CV
Female (*n* = 29)					
Weight (kg)	542	79	435	687	14.6
Age (years)	9	3	5	21	33.3
PCV (%)	35	6	30	55	17.1
TP (g/L)	68	6	62	83	8.8
Male (*n* = 21)					
Weight (kg)	561	70	434	624	12.5
Age (years)	16	13	6	23	81.3
PCV (%)	34	5	28	44	14.7
TP (g/L)	69	2	64	76	2.9

IQR: interquartile range, CV: Coefficient of variation.

**Table 2:** Total number of successful readings and percentage number of successful readings for 50 horses following three interventions.

Treatments	Post Clipping		Post Prepping		Post Medetomidine	
	(PC)		(PP)		(PM)	
Muscle of StO_2_ Probe Placement	No. Success	% Success	No. Success	% Success	No. Success	% Success
Sartorius	83/150	55	107/150	72 *	119/150	79
Biceps Brachii	49/150	33	67/135	50 *	50/150	33 ^#^
Semimembranosus	36/150	24	54/135	40 *	48/150	32
Extensor digitorum Longus	7/150	5	16/135	11	39/150	26 ^#^
Extensor Carpi Radialis	72/150	48	88/135	65 *	84/150	56
Brachiocephalicus	43/150	29	66/135	48 *	65/150	43

PC: StO_2_ values attained following clipping of hair over corresponding muscle; PP: StO_2_ values attained post clipping and cleaning with chlorhexidine of the probe placement site; PM: StO_2_ values following the clipping, cleaning and 5 min post administration of medetomidine hydrochloride 7 mcg kg^−1^ IV. Muscle: represents all the StO_2_ values attained for that muscle StO_2_ probe placement site. * Represents statistically significant differences in number of successful readings between PC and PP groups. ^#^ Represents statistically significant differences in number of successful StO_2_ readings.

**Table 3:** Summary statistics for tissue oxygen saturation (StO_2_%), measured on different muscles in 50 horses following 3 interventions.

Muscle Group and Treatment	Summary Statistics				
	Mean (90% CI)	SD	Min	Max	CV
Biceps Brachii					
Post Clipping (PC)	47 (43–51)	11	28	62	22.4
Post Prepping (PP)	46 (42–49)	10	22	63	22.7
Post Medetomidine (PM)	37 * (33–39)	10	15	45	25.7
Brachiocephalicus					
PC	46 (40–50)	12	15	67	26.9
PP	43 (40–47)	10	19	61	22.9
PM	34 * (31–37)	8	15	54	24.1
Extensor Carpi Radialis					
PC	39 (36–42)	8	26	62	21.3
PP	40 (37–43)	9	19	58	23.5
PM	30 * (28–33)	7	16	43	23.6
Extensor Digitorum Longus					
PC	38 (25–51)	11	23	48	28.1
PP	41 (36–44)	6	34	49	13.9
PM	29 (20–42)	13	16	54	47.1
Semimembranosus					
PC	45 (41–51)	10	28	67	26.9
PP	40 (35–43)	10	24	59	25.5
PM	32 * (30–36)	8	19	46	22.6
Sartorius					
PC	48 (45–51)	10	25	71	20.8
PP	48 (46–51)	9	28	70	19.6
PM	40 * (39–42)	7	20	54	16.7

* Represents statistically significant difference (*p* < 0.05) between the PP and PM interventions. Treatment: PC: StO_2_ values attained following clipping but no cleaning or preparation of the skin; PP: StO_2_ values attained post clipping and cleaning of the probe placement site; PM: StO_2_ values following the clipping, cleaning, and administration of medetomidine hydrochloride. Muscle Group: represents all StO_2_ values attained for that muscle; CV: coefficient of variance.

**Table 4:** Linear Mixed Effects Model for assessing the effect of treatment and muscle group on tissue oxygen saturation values.

Variable	Category	Estimate	SE	*t* Value	*p*-Value	95% CI	
						LCL	UCL
Intercept		47.5	5	9.6	<0.001	37.7	57.2
Treatment	PC	Ref					
	PP	0.3	0.9	0.37	0.712	−1.4	2.1
	PM	−9.2	0.9	−10.2	<0.001	−11	−7.4
Muscle group	Biceps Brachii	Ref					
	Brachiocephalicus	−2.9	1.3	−2.3	0.021	−5.4	−0.5
	Extensor Carpi Radialis	−5.6	1.1	−4.7	<0.001	−8.0	−3.3
	Extensor Digitorum Longus	−8.4	2	−4.2	<0.001	−12.3	−4.6
	Sartorius	5.0	1.2	4.2	<0.001	2.7	7.3
	Semimembranosus	−5.7	1.4	−4.2	<0.001	−8.4	−3.1
Random Effects							
σ^2^		50.18					
VarHORSE		38.25					
ICC		0.43					
NHORSE		49					
Observations		403					
Marginal R2/Conditional R2		0.350/0.631					

SE: Standard Error; CI: Confidence Interval; LCL: Lower Control Limit; UCL: Upper Control limit. VarHORSE_:_ Variance due to horse-to-horse variation, σ^2^: residual variance; ICC: Intraclass Correlation Coefficient. NHORSE: the number of horses included in the model. Marginal R2: the proportion of variance explained by the fixed effects in the model. Conditional R2: the proportion of variance explained by the random effects model. Treatment: PC: StO_2_ values attained following clipping but no cleaning or preparation of the skin; PP: StO_2_ values attained post clipping and cleaning of the probe placement site; PM: StO_2_ values following the clipping, cleaning, and administration of medetomidine hydrochloride.

## Data Availability

Datasets analyzed in this study are available upon request from the corresponding author. The data are stored on UQRDM, and mediated access is available upon request.
